# Probing the Effect of Human Normal Sperm Morphology Rate on Cycle Outcomes and Assisted Reproductive Methods Selection

**DOI:** 10.1371/journal.pone.0113392

**Published:** 2014-11-20

**Authors:** Bo Li, Yefei Ma, Jianlei Huang, Xifeng Xiao, Li Li, Chuang Liu, Yongqian Shi, Dong Wang, Xiaohong Wang

**Affiliations:** Department of Obstetrics and Gynecology, Tangdu Hospital, the Fourth Military Medical University, Xi'an, 710038, China; University of Nevada School of Medicine, United States of America

## Abstract

Sperm morphology is the best predictor of fertilization potential, and the critical predictive information for supporting assisted reproductive methods selection. Given its important predictive value and the declining reality of semen quality in recent years, the threshold of normal sperm morphology rate (NSMR) is being constantly corrected and controversial, from the 4^th^ edition (14%) to the 5^th^ version (4%). We retrospectively analyzed 4756 cases of infertility patients treated with conventional-IVF(c-IVF) or ICSI, which were divided into three groups according to NSMR: ≥14%, 4%–14% and <4%. Here, we demonstrate that, with decrease in NSMR(≥14%, 4%–14%, <4%), in the c-IVF group, the rate of fertilization, normal fertilization, high-quality embryo, multi-pregnancy and birth weight of twins gradually decreased significantly (P<0.05), while the miscarriage rate was significantly increased (p<0.01) and implantation rate, clinical pregnancy rate, ectopic pregnancy rate, preterm birth rate, live birth rate, sex ratio, and birth weight(Singleton) showed no significant change. In the ICSI group, with decrease in NSMR (≥14%, 4%–14%, <4%), high-quality embryo rate, multi-pregnancy rate and birth weight of twins were gradually decreased significantly (p<0.05), while other parameters had no significant difference. Considering the clinical assisted methods selection, in the NFMR ≥14% group, normal fertilization rate of c-IVF was significantly higher than the ICSI group (P<0.05), in the 4%–14% group, birth weight (twins) of c-IVF were significantly higher than the ICSI group, in the <4% group, miscarriage of IVF was significantly higher than the ICSI group. Therefore, we conclude that NSMR is positively related to embryo reproductive potential, and when NSMR<4% (5^th^ edition), ICSI should be considered first, while the NSMR≥4%, c-IVF assisted reproduction might be preferred.

## Introduction

Fifteen percent of couples are subfertile, and in one-half of these cases subfertility can be attributed to the male partner [1], [2]. Despite routine application, semen analysis does not provide a direct measure of functional capacity of sperm. No single semen parameter definitely predicts the potential of an individual couple to achieve success with assisted reproduction [3], however the percentage of sperm with normal morphology is positively correlated with IVF fertilization and pregnancy rates [4], [5]. Additionally sperm morphology has been demonstrated to be a better predictor of fertilization potential than other semen analysis parameter, i.e., motility and concentration, both in vivo [6] and in vitro [7], suggesting that assessment of sperm morphology represents a valuable tool with which to support clinical decisions. Given the important predictive value of sperm morphology and the the decline in semen quality reported in recent years, WHO's definition of teratozoospermia is frequently corrected. The reference value for normal sperm morphology rate (NSMR) proposed was <30% in 1992, <14% in 1999 and <4% in 2010. Due to these substantial increases in the reference values, the appropriate threshold to apply is uncertain [8].

It is often unclear whether conventional IVF or ICSI would be the most appropriate reproductive to apply, a din the case of isolated teratozoospermia, there are no widely accepted criteria governing this decision. As a result coupled with isolated teratozoospermia often pursue ICSI unnecessarily or experience complete fertilization failure following conventional IVF (c-IVF). To date, most researches on sperm morphology have focused on the correlation between NSMR and DNA integrity and chromosomal aneuploidy [9], [10], as well as its predictive value for embryonic development potential and clinical pregnancy outcomes [11], [12]. This work has produced some apparently conflicting results as samples meeting the inclusion criteria of isolated teratozoospermia are too few, or because the assessing indicators are not all around, complicating interpretation and clinical application. Moreover, very few studies reported differences in embryo quality and miscarriage rate between patients with teratozoospermia receiving c-IVF and those receiving ICSI [13].

So, this study selected 4,765 pairs of infertile couples (3922 pairs receiving c-IVF, and 843 receiving ICSI) and recorded male partner sperm morphology. Patients were further divided into three groups of NSMR ≥14%, 4%–14%, <4%. Thirteen indicators of embryo preimplantation development, clinical pregnancy outcomes, neonatal conditions were recorded in order to clarify the predictive value of NSMR. The predictive value of NSMR in c-IVF and ICSI were compared in order to provide a guidance for selecting an appropriate assisted reproductive methods for patients with teratozoospermia.

## Results

### The correlation between normal sperm morphology rate and cycle outcomes

In order to further probe the effect of human normal sperm morphology rate, 4765 pairs of infertile couples (3922 pairs receiving c-IVF, and 843 receiving ICSI) were enrolled at the Tangdu Reproductive Medical Center between January 2011 and March 2013. Patients were divided into three groups of NSMR ≥14% (the threshold was recommend by WHO^4th^), 4%–14% and <4% (the threshold was recommend by WHO^5th^). The outcome of c-IVF or ICSI - pre-implantation development, clinical pregnancy outcome and neonatal conditions – was retrospectively analyzed. There was no significant difference in male age, female age, endometrial thickness, number of oocytes retrieved, and number of embryos transferred between the patient subgroups (p>0.05) (Table 1).

**Table 1:** General description of the individuals who participated in this study.

	Normal Sperm Morphology Rate(NSMR)					
	≥14%		[4%–14%)		<4%	
	c-IVF	ICSI	c-IVF	ICSI	c-IVF	ICSI
Male age (Year)	33.36±5.59	34.87±5.05	33.45±5.36	34.58±5.12	33.36±5.9	33.21±6.3
Female age (Year)	31.52±4.53	32.12±4.78	31.54±4.68	32.07±5.01	31.59±4.92	31.24±4.82
Endometrial thickness (cm)	1.03±0.16	1.07±0.16	1.01±0.14	1.03±0.16	1.04±0.18	1.04±0.14
Oocytes retrieved (n)	12.75±5.81	11.88±6.1	12.59±5.73	11.51±5.85	12.74±5.67	12.48±6.14
Embryos transferred (n)	2.37±0.49	2.4±0.63	2.28±0.52	2.22±0.65	2.18±0.56	2.14±0.58

Values are mean ± SD.

**Figure 1:**
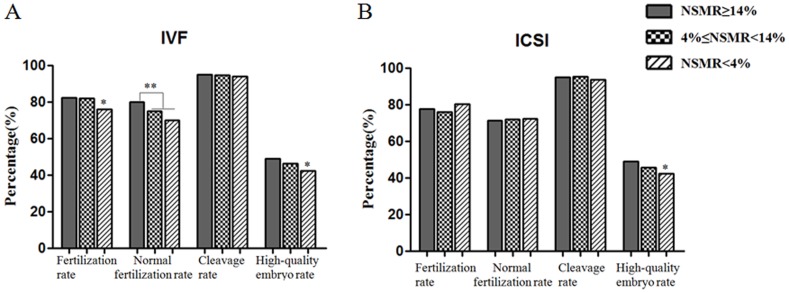
The relationship between sperm morphology and preimplantation embryo development. A. The relationship between the NSMR and embryo potential originated from c-IVF cycle. B. The relationship between the NSMR and embryo potential originated from ICSI cycle. Values are n (%), *P<0.05, **P<0.01. NSMR: Normal Sperm Morphology Rate.

During preimplantation development, we retrospectively analyzed four indicators (fertilization rate, normal fertilization rate, cleavage rate, high quality embryo rate). Results showed that in the c-IVF treatment group, with the decrease in NSMR (≥14%, 4%–14% and <4%, the same below), fertilization rate, normal fertilization rate and high quality embryo rate gradually decreased, and the decrease reached a statistical significance or very significance (P<0.05 or p<0.01, Figure 1A), while cleavage rate remained at a high level, ranging from 94.03% to 95.01%, without any significant difference (P>0.05). In the ICSI treatment group, with the decrease in NSMR, fertilization rate and normal fertilization rate showed no regular changes, and the difference is not statistically significant (p>0.05, Figure 1B), however, the high quality embryo rate gradually decreased significantly (P<0.05). Similar to the c-IVF group, cleavage rate maintained at a high level in each subgroup, ranging from 93.69% to 95.45%, without any statistically significant difference (P>0.05).

**Figure 2:**
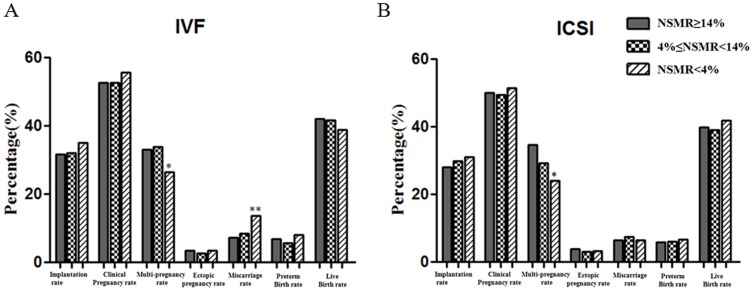
The relationship between sperm morphology and clinical outcomes. A. The relationship between the NSMR and c-IVF clinical outcomes; B. The relationship between the NSMR and ICSI clinical outcomes. Values are n (%), **P<0.01. NSMR: Normal Sperm Morphology Rate.

We retrospectively analyzed seven indicators of clinical pregnancy outcome, including implantation rate, clinical pregnancy rate, multiple pregnancy rate, ectopic pregnancy rate, miscarriage rate, premature birth rate, and live birth rate. In the c-IVF treatment groups, with the decrease in NSMR, implantation rate, clinical pregnancy rate, ectopic pregnancy rate, premature birth rate, and live birth rate showed no significant difference, however, miscarriage rate in the NSMR <4% sub-group was significantly higher (p<0.01, Figure 2A) compared with each other subgroups. In the ICSI treatment group, NSMR had no significant correlation with implantation rate, clinical pregnancy rate, ectopic pregnancy rate, miscarriage rate, premature birth rate and live birth rate (P>0.05). In addition, both groups in the c-IVF and ICSI treatment, multiple pregnancy rate showed a gradual downward trend with the decrease in NSMR, and the difference was significant (P<0.05, Figure 2A).

**Figure 3:**
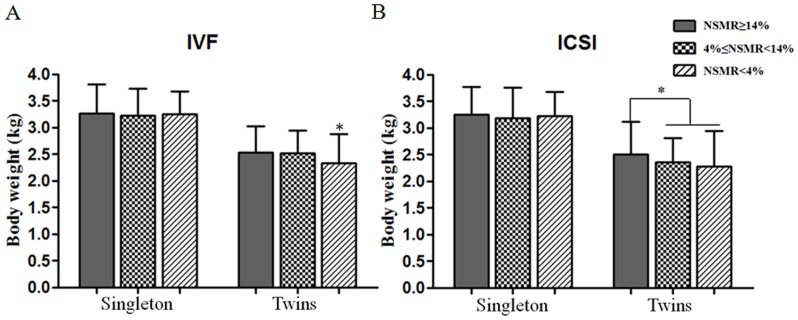
The relationship between sperm morphology and body weight. A. The relationship between the NSMR and body weight from c-IVF cycle. B. The relationship between the NSMR and body weight from ICSI cycle. Values are mean ±SD, * P<0.05. NSMR: Normal Sperm Morphology Rate.

We retrospectively analyzed two indicators of neonatal conditions, sex ratio and neonatal birth weight of singletons and twins. From Table 2 we can see that, except the two NSMR ≥14% and NSMR <4% subgroups in ICSI treatment group showing a male to female ratio of 0.97 and 1.01, the other subgroups showed a higher proportion of boys. Birth weight was analyzed in singletons and twins separately. Results showed that the birth weight of singleton in both the c-IVF group and ICSI group fluctuated between 3.18±0.58 kg to 3.27±0.55 kg, without any statistically significant difference (P>0.05, Figure 3), while the birth weight of twin, whether in the c-IVF or ICSI group, showed a gradual downward trend, with statistically significant difference (p<0.05, Figure 3).

**Table 2:** The indicative effect of NSMR on decision making of clinical assisted reproductive method selection (c-IVF/ICSI).

	Normal Sperm Morphology Rate(NSMR)					
	≥14%		[4%–14%)		<4%	
	c-IVF	ICSI	c-IVF	ICSI	c-IVF	ICSI
Cycle number (n)	1247	152	2450	303	225	388
Fertility rate (%)	82.42	77.75	82.13	76.07	76.05	80.22
Normal fertility rate (%)	79.91	71.40*	75.08	71.88	69.97	76.32
Cleavage rate (%)	95.01	94.91	94.76	95.45	94.03	93.69
High quality embryo rate (%)	49.08	48.86	46.38	45.51	42.46	42.24
Implantation rate (%)	31.5	28	31.99	29.7	34.99	31.08
Clinical pregnant rate (%)	52.6	50	52.69	49.45	55.69	51.31
Multipul pregnant rate (%)	32.93	34.62	33.77	29.19	26.34	24.06
Ectopic pregnant rate (%)	3.45	3.85	2.56	3.05	3.36	3.12
Miscarriage rate (%)	7.13	6.35	8.44	7.36	13.6	6.32**
Preterm birth rate (%)	6.82	5.77	5.67	5.91	7.95	6.52
Live birth rate (%)	42.02	39.8	41.67	39.04	38.73	41.87
Sex ratio (M/F)	1.13	0.97	1.1	1.22	1.32	1.01
Birth weight (kg, Twins)	2.54±0.49	2.50±0.62	2.52±0.42	2.36±0.46*	2.33±0.55	2.28±0.66
Birth weight (kg, Singleton)	3.27±0.55	3.35±0.53	3.22±0.51	3.18±0.58	3.25±0.43	3.23±0.45

Values are mean ±SD, n or n (%),

*refers to ICSI cycle outcomes compared with c-IVF outcomes in the same subgroup of NSMR, P<0.05,

**denote P<0.01.

### Selection of assisted reproductive methods based on normal sperm morphology rate

Further, we analyzed the influence of c-IVF or ICSI on embryo development potential and clinical outcome in patients with different normal sperm morphology rates. From Table 2 we can see that in the NSMR ≥14% subgroup, c-IVF and ICSI assisted reproduction had no significant difference with regard to fertilization rate, cleavage rate, high quality embryo rate, implantation rate, clinical pregnancy rate, multiple pregnancy rate, ectopic pregnancy rate, miscarriage rate, preterm birth rate, live birth rate, sex ratio, and birth weight in singletons and twins(p>0.05), but in normal fertilization rate, c-IVF treatment group were significantly better than ICSI group(79.91 Vs 71.40, p<0.05). Therefore, for couples with NSMR ≥14%, c-IVF assisted reproductive method can significantly improve utilization efficiency of embryos.

In the 4%≤NSMR<14% subgroup, c-IVF and ICSI assisted reproduction had no significant difference in the aspects of fertilization rate, normal fertilization rate, cleavage rate, high quality embryo rate, implantation rate, clinical pregnancy rate, multiple pregnancy rate, ectopic pregnancy rate, miscarriage rate, preterm birth rate, live birth rate, sex ratio, and birth weight of singletons (p>0.05). However, ICSI reproduction had lower birth weight of twins than c-IVF group, with statistically significant difference (2.36±0.46 Vs 2.52±0.42, p<0.05). Therefore, in couples with 4%≤NSMR<14%, c-IVF assisted reproductive method is preferred.

In the NSMR <4% subgroup, ICSI assisted reproduction had miscarriage rate of 6.32%, while c-IVF assisted reproduction had miscarriage rate of 13.6%. The two had statistically significant difference (p<0.01). Therefore, for infertile couples with NSMR<4%, ICSI assisted reproductive method is preferred to reduce abortion rate.

## Discussion

To the best of our knowledge, this is a first study to analyze the relationship between the sperm morphology and the clinical selection of assisted reproductive methods based on comprehensive parameters, from the preimplantation to newborn stage. And, a preliminary view is that for patients with NSMR <4%, ICSI assisted reproductive method should be given in priority, and for patients with NSMR≥4%, c-IVF assisted reproductive method should be chosen in priority.

Some studies have demonstrated that fertilization potential appears to decline with advancing paternal age. Ford et al. [14] reported that increasing paternal age is associated with delayed conception in a large population of fertile couples. In a large French study, Belloc et al. [15] analyzed and reported a miscarriage rate of 13.7% per pregnancy in men <30 years versus 32.4% in men ≥45 years (P<0.001). And in a prospective study of 221 couples undergoing c- IVF, Klonoff-Cohen et al. reported a 38% live birth rate for men <35 years, and only 7% for men >40 years (P<0.01). Additionally, women with thyroid dysfunction [16] will result in decreased implantation rate and clinical pregnancy rate after assisted conception, as well as increased abortion rate and intrauterine fetus growth restriction. Moreover, women with uterine operation histories had a higher ratio of ectopic pregnancy occurrence. Therefore, in this study, in order to avoid confounding factors, we had a strict control over the above conditions, and our data showed that patients enrolled had no significant differences in endometrial thickness, number of oocytes retrieved, and number of embryos transferred, ensuring the maximum reliability of the results in our study.

In our sample, in patients that received c-IVF assisted conception the embryo fertilization rate and normal fertilization rate gradually declined with NSMR, however, these measured were not correlated with NSMR in patients who received ICSI assisted conception. The most likely explanation for this is that during ICSI the embryologist can microscopically select individual sperm that appear morphologically “normal,” from even the most impaired specimens. Thus, fertilization occurs with sperm that may not be representative of the sperm population within the entire sample, making the initial semen morphology assessment irrelevant. This concept has been taken to the subcellular level in new techniques such as intracytoplasmic morphologically selected sperm injection and motile sperm organellar morphology examination (MSOME). In these techniques, investigators select sperm for ICSI using a high power×63,000 objective to magnify and allow more critical assessment of sperm morphology. Sperm nucleus morphology by the MSOME method has positively correlated with fertilization and pregnancy rates [17]. In addition, our data also showed that regardless of the degree of NSMR, no matter what (c-IVF or ICSI) assisted reproductive method was adopted, the embryo cleavage rate would not be affected, but the rate of high quality embryo would gradually decrease with the decrease in NSMR. On one hand, this showed that the current embryo quality assessing criteria has a good reference value, which is consistent with the results of Terriou et al [18]. Their study suggested that embryo score was a good predictor of embryo quality and pregnancy rates. On the other hand, it showed that the predictive value of assessing indicators used in our laboratory before embryo implantation is different. Maybe we should weigh the importance of these indicators (high quality embryo rate> normal fertilization rate> fertilization rate> cleavage rate) for a more precise assessment on the embryo qualities.

In clinical practice, we select the best quality embryos for transplantation. This step of evaluation largely offsets the impact of paternal abnormal sperm on outcome.. Therefore, regardless of the degree of sperm normal morphology, and method of reproductive technology employed, the embryo implantation rate, clinical pregnancy rate, ectopic pregnancy rate, and premature birth rate did not differ between patients subdivided by NSMR.

However, in contrast, we did find that the rate of multiple births decreased gradually with the decrease in NSMR, regardless of the method of insemination, reflecting the important contribution of male factors on embryonic development potential. We were also surprised to find that, when using c-IVF, in patients with NSMR <4%, miscarriage rate increased, and live birth rate decreased. Reasons may be that, although c-IVF retained the natural property of sperm-egg recognition, combination and penetration and is closer to a physiological insemination, compared to the normal process of fertilization, it lacks the screening mechanisms of PH value, antibody killing, and liquid concentration gradient on sperms in the “trek” process from the cervical to the oviduct belly. Therefore, when semen with a lower NSMR is used for c-IVF insemination, it means that there is a great probability for abnormal sperms to combine with eggs and fertilize, while in the normal fertilization process, this part of abnormal sperms may not be able to reach the ampulla.

Previous studies have indicated that, in comparison to other parameters of sperm, sperm morphology is a more reliable predictor of clinical outcomes. Lewis-Jones et al [19] clearly showed in their study that NSMR was closely related to chromosomal abnormalities. In patients with severe teratozoospermia [20], and patients with oligozoospermia or azoospermia [21], sperm DNA fragmentation and degree of aneuploidy were significantly higher, and a higher percentage of aneuploidy was also found in embryos [22]. Further studies showed that even these aneuploidy sperms could fertilize successfully to conceive [23], but the early term spontaneous abortion rates would be significantly higher [24], which was consistent with the results of our analysis. However, in the NSMR <4% group, when using ICSI assisted conception, regardless of the degree of NSMR, miscarriage rate was not significantly affected (P>0.05). This might be because that prior to ICSI assistant reproduction, there was an additional filtering mechanism on sperms. Through artificial selection by embryologists, the chance of fertilization of sperms with morphological abnormalities were largely ruled out, and therefore, after microinjection in patients with different normal sperm morphology rates, the miscarriage rate would had no significant correlation with the NSMR, without any significant difference in each group. This explanation was further confirmed by Antinori et al [25] in their report- abnormal sperm screening in teratozoospermia patients significantly reduces abortion rate.

Male and female preimplantation embryos differ not only in their chromosomal complement, but in their proteome and subsequent metabolome. Studies have reported [26], [27] that embryos of different sex have different metabolic levels for amino acids and glucose. Furthermore, on Day 4 female embryos consumed 28% more glucose compared with males (P<0.05). Interestingly, the presence of glucose during in vitro culture inhibits the development of female embryos more than that of the male counterparts [28], indicating a selective embryotoxicity towards females. But rather, the rate of embryonic development is a very important indicator in our assessment of embryo quality. Therefore, the elevated newborn male/female ratio in c-IVF/ICSI appeared in the present study may be related to our early embryo evaluation and selection criteria.

With the widespread application of assisted reproductive technology more potential parents express concerns about potential birth defects. Most studies [29], [30] believe that ART children had lower birth weights, but the exact causes and molecular mechanisms are not yet known. Shih et al [29] suggested that the phenomenon of low birth weight is not significantly related to ART technology itself, nor clearly related with the in vitro process [30]. Our results showed that for singleton infants, c-IVF and ICSI had no significant difference in birth weight, while for twins, with the decrease in NSMR, both methods showed a gradual downward trend in birth weight (p<0.05), reminding us that sperm factors play an important role in this. Mouse male parthenogenesis model has proven expressing of paternal genes affects the formation and growth of placenta to maintain the normal structure and function of the placenta [31]. If mouse eggs after parthenogenetic activation are implanted into surrogate maternal womb, embryonic development will be stopped after 10.5 days due to developmental placenta dysplasia. A previous study in mice [32] suggested that the phenomenon of low birth weight was related with the decrease in efficiency of placental nutrient supply. Haeussner et al [33] also made it clear that birth weight was closely related to the size of placenta. There are many imprinting genes in human placenta involved in the regulation of fetal-maternal substance transport, affecting the growth and development of the placenta and fetal, such as H19, and IGF2, etc. Deletion or abnormal activation of these genes can lead to fetal growth abnormalities [34]. Studies have shown [35] that after the H19 gene in one of the chromosomes in parthenogenetic mouse embryo was knocked out to simulate paternal imprinting, the parthenogenetic embryo could develop normally and successfully give birth to healthy baby mice. Yu et al [36] suggested that in early pregnancy once DNA methylation in H19-ICR region was lost, resulting in H19 over expression, it would cause trophoblast differentiation disorder and promote trophoblast apoptosis, resulting in disruption of embryonic development and abortion, etc., and indeed in ICSI human placenta, DNA methylation in H19-ICR region was found to be significantly lower [37]. In addition, many studies have shown that in the sperms of patients with oligozoospermia, azoospermia and teratospermia, H19 [38], DAZL [39] and other genes had abnormal DNA methylation levels, and in abnormal sperms after density gradient centrifugation, the abnormal DNA methylation levels were more significant. Higher sperm deformity rate may lead to a higher chance of abnormal expression of imprinting genes, causing inefficient placental nutrient supply, which is more prominent in multiple pregnancies, resulting in significant reduction in birth weight of twins.

In conclusion, our study suggests that NSMR is closely related to the potential of embryonic development and cycle outcomes, and can serve as a powerful reference in clinical choice of assisted reproductive methods in patients with teratozoospermia. The molecular mechanisms governing these observations remaind to be determined, and we recommend genetic and epigenetic analysis of the embryo and placenta in teratozoospermia patients.

## Materials and Methods

### Patients

The c-IVF and ICSI cycles were performed at the Tangdu reproductive medical center from January 2011 to March 2013 and retrospectively analyzed. The inclusion criteria of infertile couples were as follows: male, age<38 years old; female, age <38 years old, infertility due to tubal factor or unexplained infertility. The exclusion criteria: patients with endometrial fibroids, endometriosis, uterine adhesion due to previous uterine surgery, or thyroid function abnormality [16] were ruled out. The written informed consent was obtained from participants. This study was approved by the local ethical committee of Tangdu hospital.

### Sperm/Semen analysis and manipulation

Semen samples were collected by masturbation for semen analysis after an abstinence of 3–5 d. In all the occasions, semen volume, concentration, and motility were determined according to 5^th^ WHO [40] criteria (semen volume ≥1.5 ml, concentration ≥15×10^6^/ml, total count ≥39×10^6^, total motility ≥40% or progressive motility ≥32%).

Sperm morphology was assessed using the Kruger/Tygerberg Strict Criteria as outlined by the 5^th^ WHO manual. The criterion used for assessment of sperm morphology: (1) The head should be smooth, regularly contoured and generally oval in shape. There should be a well-defined acrosomal region comprising 40–70% of the head area. The acrosomal region should contain no large vacuoles, and not more than two small vacuoles, which should not occupy more than 20% of the sperm head. The post-acrosomal region should not contain any vacuoles. (2) The midpiece should be slender, regular and about the same length as the sperm head. The major axis of the midpiece should be aligned with the major axis of the sperm head. Residual cytoplasm is considered an anomaly only when in excess, i.e. when it exceeds one third of the sperm head size. (3) The principal piece should have a uniform calibre along its length, be thinner than the midpiece, and be approximately 45um long (about 10 times the head length). It may be looped back on itself, provided there is no sharp angle indicative of a flagellar break. Basic Procedure is as follow: Briefly, 5–10 µl of semen (depending on the sperm concentration) was placed on a previously cleaned slide and stained using the Papanicolaou staining protocol. Two technicians, who have gotten the national docimaster qualification and have been trained for documenting teratospermia with the standard of WHO-5, analyzed at least 200 sperm cells independently under oil immersion microscope with 1000× magnification. Mean values of their reports were recorded. Quality control mechanism for morphology assessment included a weekly calculation of inter-observer coefficient of variation as obtained by their concurrent evaluation of the same discarded semen sample. An inter-observer variation of <5% was considered to be acceptable. The dyes were checked daily for cross contamination and were changed weekly.

### IVF/ICSI Procedure

Down-regulation with leuprolide acetate (Lupron; TAP Pharmaceuticals, Lake Forest, IL) followed by ovulation induction with gonadotropins was the primary stimulation protocol used. Oocytes were recovered by transvaginal aspiration of follicles under ultrasound guidance. Oocytes were cultured in microdrops of human tubal fluid(HTF) medium(Life Global, Guilford, CT) supplemented with 10 mg/ml human serum albumin(Cooper Surgical, Trumball, CT) under an oil overlay. Culture dishes were incubated at 37°C with 6% CO_2_. Oocytes were placed in 15,000 sperm per 100-ul drop or fertilized by ICSI 3–4 h after the retrieval. Fertilization check was performed 18–20 h after the c-IVF/ICSI procedure. Normally fertilized zygotes were subsequently moved to microdrops containing HTF medium with 10% synthetic serum substitute(SSS, Irvine, CA) and cultured for an additional 2 days. Embryo transfer was performed on day 3 under ultrasound guidance using a Wallace catheter. Embryo selection for day 3 transfer was based on morphologic parameters. Embryos were graded on the basis of cell number, regularity of blastomeres, good blastomere expansion, fragmentation level, and signs of embryonic compaction. Pregnancy testing was performed 15 days after the embryo transfer. Clinical pregnancy was confirmed by the presence of a fetal heart on ultrasonic examination at 6–8 weeks of pregnancy.

### Statistical analysis

The rates of fertilization, normal fertilization, cleavage, high quality embryo, implantation, pregnancy, multiple pregnancy, ectopic pregnancy, miscarriage, preterm birth, live birth and sex ratio were compared between groups using chi-square analyses. Female age, male age, endometrial thickness, number of oocytes retrieved, number of embryos transferred and birth weight were compared between groups using Student's t test. P values of <0.05 were considered to be statistically significant.

## Supporting Information

Figure S1 **The basic information of patient couple treated by c-IVF.**
(XLSX)

Figure S2 **The basic information of patient couple treated by ICSI.**
(XLSX)
